# A Nemertean-Derived Peptide Toxin Exhibits Oral Insecticidal Activity Against *Spodoptera litura*

**DOI:** 10.3390/md24090310

**Published:** 2026-09-04

**Authors:** Wenxian Wu, Yueyue Liu, Qipeng Jiang, Yi Cai, Xingyue Li

**Affiliations:** 1 Institute of Urban Agriculture, Chengdu Agricultural Science and Technology Center, Chinese Academy of Agricultural Sciences, Chengdu 610299, China; wuwenxian@caas.cn (W.W.); jiangqipeng@caas.cn (Q.J.); 2 Sichuan Academy of Agricultural Sciences, Chengdu 610066, China; yueyueliu@scsaas.cn; 3 College of Life Science, Sichuan Agricultural University, Ya’an 625000, China

**Keywords:** *Spodoptera litura*, nemertide α-1, marine peptide toxin, oral insecticidal activity, peptide bioinsecticide

## Abstract

*Spodoptera litura* is a polyphagous lepidopteran pest with rapidly evolving insecticide resistance. Peptide toxins from venomous organisms, many stabilized by the inhibitor cystine knot motif, offer bioinsecticidal potential but show limited oral efficacy. Seven previously reported candidates were recombinantly produced in *Pichia pastoris* and screened against *S. litura* by injection and leaf-disc feeding bioassays, followed by whole-plant spray assays. The marine nemertean toxin nemertide α-1, designated A1, showed the strongest oral activity, with a median lethal concentration of 3.97 μg μL^−1^. Conversely, the engineered spider-venom peptide U1-AGTX-Ta1b R9Q (T1) was most toxic by injection but showed no detectable oral activity, demonstrating that injection toxicity did not predict oral efficacy. High-cell-density fermentation yielded an estimated A1 titer of 1.20 g L^−1^. Whole-plant foliar application of A1 showed concentration-dependent efficacy. Mortality reached 92.07% at 8 μg μL^−1^, whereas lower concentrations suppressed larval growth, reducing the mean weight of surviving larvae by 41.55% and 67.58% at 1 and 2 μg μL^−1^, respectively, and mitigated feeding damage. These findings extend the documented oral insecticidal spectrum of nemertide α-1 to *S. litura* and demonstrate whole-plant efficacy following foliar application, supporting its development as an active ingredient for foliar-applied peptide bioinsecticides.

## 1. Introduction

The tobacco cutworm, *Spodoptera litura* (Fabricius) (Lepidoptera: Noctuidae), is a destructive polyphagous pest distributed throughout tropical and subtropical regions of Asia and Oceania [1]. Its larvae attack more than 100 economically important crops across diverse plant families, including Brassicaceae, Solanaceae, Fabaceae, and Poaceae [2]. During outbreaks, late-instar larvae feed voraciously, rapidly consuming foliage and young stems while also damaging flowers and developing fruits, resulting in substantial yield and economic losses. High fecundity and overlapping generations facilitate rapid population growth, whereas intensive insecticide use has selected for resistance to multiple chemical classes, compromising control efficacy and complicating field management [2,3]. These challenges underscore the need for environmentally compatible control agents that act on molecular targets distinct from those of currently used compounds.

Venoms and defensive secretions from spiders, scorpions, parasitoid wasps, and numerous marine invertebrates constitute rich reservoirs of insecticidal peptides [4,5]. Many of these molecules are stabilized by multiple disulfide bonds, with toxins adopting an inhibitor cystine knot (ICK) motif representing a prominent structural class [6]. They frequently act on voltage-gated ion channels or other neuronal receptors, disrupting signal transmission and ultimately inducing paralysis or death [7]. For example, β-diguetoxin-Dc1a from the desert bush spider *Diguetia canities* binds the voltage sensor in domain II of insect voltage-gated sodium channels and stabilizes its activated conformation, thereby favoring channel opening while displaying pronounced insect selectivity [8]. Other insecticidal peptides compromise membrane integrity or interfere with protein synthesis and intracellular signaling [5]. Their conformational stability, mechanistic diversity, and high biological activity make these molecules attractive candidates for peptide-based bioinsecticide development [7].

Despite these advantages, the poor oral efficacy of many venom-derived peptides remains a major obstacle to their use as foliar insecticides. Because venom peptides generally evolved for direct delivery into prey tissues, they may have experienced limited selective pressure to withstand the digestive environment or traverse the midgut epithelium. Consequently, marked toxicity following injection often fails to translate into comparable activity after ingestion [9]. Proteolysis by digestive enzymes, restricted epithelial permeability, inefficient transepithelial transport, and rapid clearance after entry into the hemocoel can all reduce the amount of intact peptide reaching its molecular target [10]. Overcoming these barriers is therefore critical for converting insecticidal peptides into effective foliar-applied agents.

Three complementary approaches have been pursued to improve oral efficacy: direct identification of naturally orally active toxins, molecular engineering to enhance proteolytic stability, and carrier-assisted transport across the midgut [9,10]. Through direct oral screening, Hardy et al. identified U1-theraphotoxin-Spl1a (Spl1a) from the venom of the Australian tarantula *Selenotypus plumipes* [11]. Spl1a exhibited an oral median lethal dose (LD_50_) of 104.2 ± 0.6 pmol g^−1^ against the cotton bollworm *Helicoverpa armigera* [11]. Davis et al. subsequently showed that U1-AGTX-Ta1b was readily cleaved by trypsin-like proteases in the lepidopteran gut and identified Arg9 as a major cleavage site [12]. Replacing this residue with glutamine enhanced proteolytic stability and conferred oral activity on the resulting R9Q variant [12]. Carrier-mediated delivery provides a complementary means of overcoming the midgut barrier [10]. Snowdrop lectin, *Galanthus nivalis* agglutinin (GNA), binds glycosylated components of the insect midgut and can promote the translocation of fused cargo across the epithelium into the hemolymph [13]. GNA has consequently been used as a carrier for several insecticidal peptides [14,15,16,17]. Although orally administered δ-amaurobitoxin-PI1a alone showed no detectable activity against cabbage moth larvae, a single 30 μg dose of the PI1a/GNA fusion protein caused 100% mortality among third-instar larvae within 6 days and significantly reduced the survival, growth, and food consumption of later instars [18]. These studies demonstrate that direct screening, molecular stabilization, and carrier-assisted delivery can each generate peptides or fusion proteins with improved oral insecticidal activity.

Evidence of oral activity also extends beyond spider toxins and lectin-based fusion proteins. LqhIT2, originally identified in the venom of the Israeli yellow scorpion *Leiurus hebraeus*, was subsequently produced recombinantly and shown to be orally toxic to fifth-instar *Pieris rapae* larvae [19]. Pinto et al. identified several ICK peptides in teratocyte secretions from the endoparasitoid wasp *Cotesia flavipes*. CftICK-I, CftICK-II, and CftICK-III suppressed hemocyte-mediated encapsulation and, following ingestion, increased mortality and reduced leaf consumption in *Diatraea saccharalis*, a permissive host of *C. flavipes*. CftICK-III elicited similar oral effects in the nonpermissive host *S. frugiperda* [20,21].

Advances in omics and mass spectrometry have further extended peptide-toxin discovery to previously understudied animal taxa. Nemerteans, commonly known as ribbon worms, are predominantly marine predators and scavengers [22]. They capture prey using an eversible proboscis, which is armed with a stylet in some lineages, while their epidermal mucus contributes to prey capture and defense. Jacobsson et al. identified a previously undescribed family of ICK toxins, termed α-nemertides, in the epidermal mucus of the bootlace worm *Lineus longissimus* [23]. Mature native nemertide α-1 comprises 31 amino acid residues and contains three disulfide bonds arranged in an ICK motif. It exerts neurotoxic effects by slowing the inactivation of insect voltage-gated sodium channels [23]. Oral insecticidal activity of recombinant nemertide α-1 has already been demonstrated against *Mamestra brassicae* larvae and the aphids *Myzus persicae* and *Acyrthosiphon pisum* [24], while more recent work documented feeding deterrence in *M. persicae* [25]. However, neither its oral insecticidal activity against *S. litura* nor its efficacy following foliar application to intact plants has been evaluated.

Comparative assessment of candidate toxins requires sufficient quantities of correctly folded recombinant products. *Escherichia coli* and yeast are among the most widely used platforms for recombinant protein production. Although *E. coli* offers rapid growth and straightforward genetic manipulation, small disulfide-rich peptides frequently misfold or accumulate in inclusion bodies during conventional cytoplasmic expression [26]. Yeast hosts provide eukaryotic folding and secretory pathways that facilitate oxidative folding and disulfide-bond formation. Secretion into the culture medium also reduces contamination by intracellular proteins and simplifies downstream purification. *Pichia pastoris* has been used successfully to produce several insecticidal peptides at high titers. Wu et al. reported a titer of 2.30 g L^−1^ for the HxTx-Hv1h/CPP-1838 fusion peptide, whereas Li et al. obtained 1.4 g L^−1^ of recombinant LqhIT2 [19,27]. These results demonstrate the suitability of *P. pastoris* for scalable production of disulfide-rich insecticidal peptides.

To identify candidates with oral insecticidal activity against *S. litura*, we selected seven previously reported molecules based on their documented insecticidal effects, oral activity in other insect species, or structural features considered potentially favorable for oral delivery (Table S1). The panel comprised five previously reported mature toxin sequences—A1, C1, D1, L1, and S1 [8,11,19,21,23,28,29]—and two previously engineered molecules. PIGA retained the published PI1a–AAA–GNA fusion architecture [18], whereas T1 retained the published R9Q substitution in U1-AGTX-Ta1b [12]. For side-by-side evaluation, DNA sequences encoding all seven candidates were codon-optimized and expressed using the same methanol-inducible secretory system in *P. pastoris*. Injection bioassays served as a first-tier functional screen to determine whether each recombinant preparation retained insecticidal activity when delivered directly to internal tissues. Candidates displaying measurable injection lethality were subsequently evaluated in leaf-disc feeding assays. The most orally active peptide was then produced by high-cell-density fermentation and assessed by foliar application to whole pepper plants. This tiered design enabled comparison of injection and oral performance among the injection-active candidates, determination of whether the previously reported oral activity of A1 extended to *S. litura*, and evaluation of the production scalability and whole-plant protective efficacy of the lead peptide.

## 2. Results

### 2.1. Recombinant Production of Seven Insecticidal Candidates

Seven previously reported insecticidal candidates were recombinantly produced in *P. pastoris*. The panel comprised five candidates corresponding to previously reported mature toxin sequences—A1, C1, D1, L1, and S1—and two candidates based on previously engineered designs, PIGA and T1 (Table S1 and Figure S1). For recombinant expression, each coding sequence was codon-optimized and inserted downstream of the α-factor secretion leader in the methanol-inducible vector pPICZαA. The expression junctions were configured so that proteolytic processing of the secretion leader would yield the intended N terminus without introducing additional vector-derived residues. A C-terminal 6×His tag was incorporated to facilitate purification by nickel-affinity chromatography.

Following linearization with *Sac* I and electroporation into *Pichia pastoris* X-33, 14 independent Zeocin-resistant transformants from each construct were screened by small-scale methanol induction. Direct SDS–PAGE analysis of the culture supernatants revealed discernible putative product bands for all seven constructs, although their intensities varied considerably among transformants (Figure S2). For each construct, the transformant displaying the most distinct and relatively intense band in the relevant molecular-mass region was selected for scale-up production.

**Figure 1:**
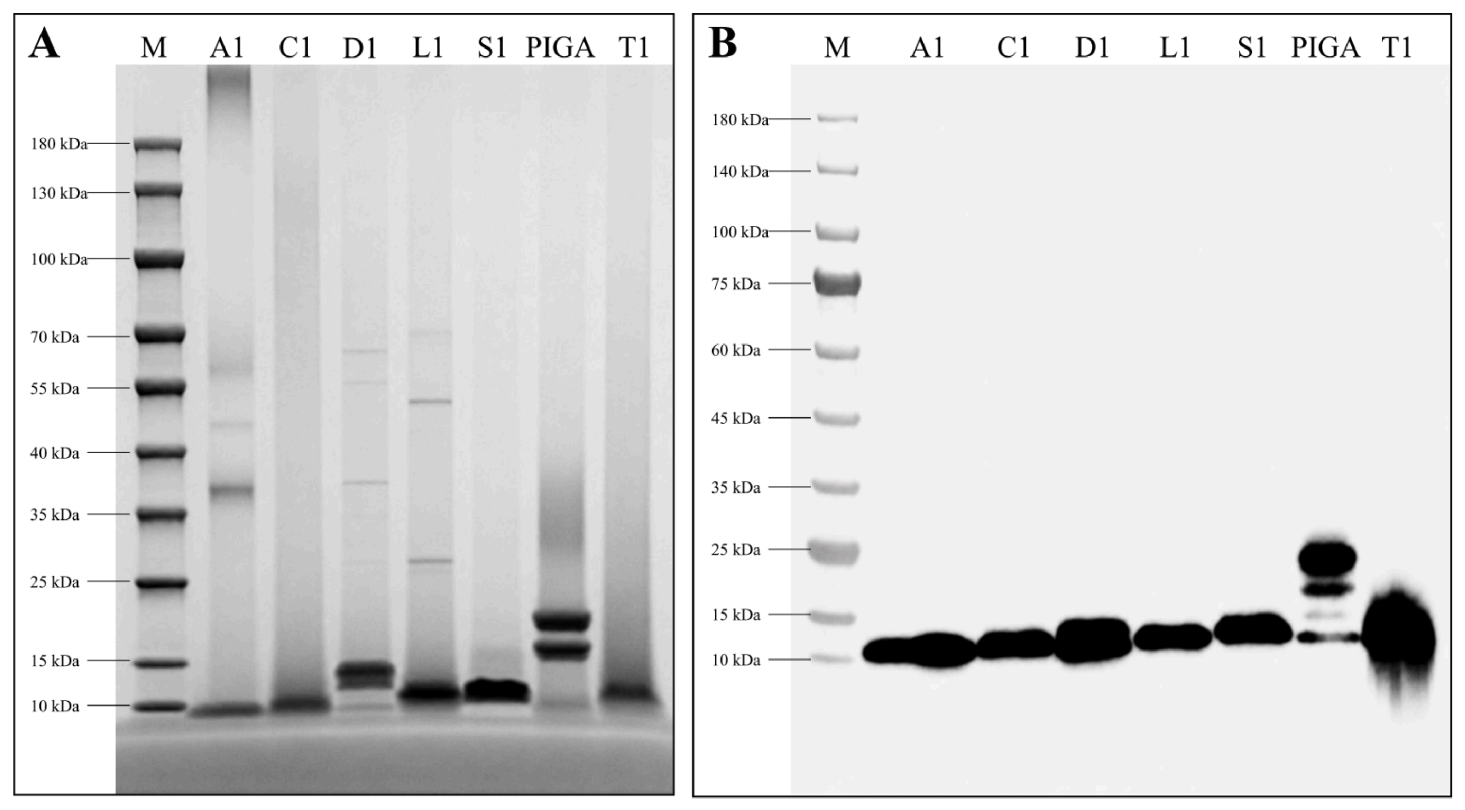
SDS-PAGE and Western blot analyses of purified recombinant products. (**A**) Coomassie Brilliant Blue-stained SDS-PAGE analysis of recombinant products purified from the culture supernatants of *P. pastoris* X-33 by nickel-affinity chromatography. (**B**) Anti-His Western blot detection of recombinant products carrying a C-terminal 6×His tag. For both analyses, 5 μg of each purified recombinant product was loaded per sample lane. M, molecular mass marker; A1, nemertide α-1; C1, CftICK-III; D1, β-diguetoxin-Dc1a; L1, LqhIT2; S1, U1-theraphotoxin-Spl1a; PIGA, PI1a/Galanthus nivalis agglutinin fusion protein; and T1, the R9Q variant of U1-AGTX-Ta1b. Molecular masses are indicated in kilodaltons (kDa).

Following preparative shake-flask induction, the secreted products were recovered from the culture supernatants by nickel-affinity chromatography. SDS–PAGE revealed predominant bands in all seven purified preparations (Figure 1A). For the six peptide toxin preparations, the principal bands were located mainly in the low-molecular-mass region and corresponded to those observed during transformant screening (Figure S2). ImageJ densitometric analysis yielded apparent electrophoretic purities of 89.3%, 96.7%, 95.0%, 95.0%, 96.3%, and 97.0% for A1, C1, D1, L1, S1, and T1, respectively. These values indicated that single-step nickel-affinity chromatography yielded the six recombinant peptide toxin preparations with relatively high apparent electrophoretic purity. Western blotting with an anti-His-tag antibody detected immunoreactive signals in every purified preparation at positions matching the principal Coomassie-stained bands (Figure 1B). Anti-His-immunoreactive signals coinciding with the corresponding SDS–PAGE bands supported the presence of C-terminally His-tagged recombinant products in the purified preparations.

The principal His-immunoreactive bands detected in all six peptide toxin preparations migrated more slowly than expected from their calculated molecular masses. Nemertide α-1 (A1), for example, had a theoretical molecular mass of approximately 4.1 kDa when its C-terminal 6×His tag was included, whereas its principal band migrated at approximately 10 kDa. A comparable migration pattern has previously been reported for recombinant nemertide α-1 produced in *P. pastoris* using the pGAPZαB vector, which also appeared at approximately 10 kDa on SDS–PAGE [24]. The L1 preparation showed a similar discrepancy, with its principal band migrating in the approximately 10-kDa region. In an earlier study, recombinant LqhIT2 produced using the same pPICZαA-based *P. pastoris* expression system displayed a comparable apparent molecular mass and was subsequently identified as LqhIT2 by nanoLC–MS/MS [19]. Upward shifts in apparent molecular mass have also been reported for other *P. pastoris*-expressed, disulfide-rich insecticidal peptides, including Hv1a and HxTx-Hv1h [24,27]. These independent observations support the interpretation that reduced electrophoretic mobility is a reproducible feature of small, cysteine-rich recombinant peptides and can account for the discrepancy between their calculated and SDS–PAGE-derived molecular masses.

PIGA exhibited a more complex electrophoretic profile. In addition to a predominant species at higher apparent molecular mass, at least one lower-molecular-mass band was detected by SDS–PAGE and recognized by the anti-His antibody (Figure 1A,B). This profile closely resembled that previously reported for PI1a/GNA expressed in *P. pastoris* [18]. In that study, N-terminal sequencing confirmed the identity and correct secretory processing of the principal fusion product, whereas PNGase F treatment caused the disappearance of an approximately 21-kDa species and higher-molecular-mass material, demonstrating the presence of glycosylated forms. The close agreement between this previously characterized profile and the multiple His-immunoreactive PIGA species observed here supports the interpretation that the electrophoretic heterogeneity of the present preparation is consistent with the established glycosylation-related behavior of yeast-expressed PI1a/GNA.

Overall, the pPICZαA methanol-inducible secretion system yielded C-terminally His-tagged preparations corresponding to all seven constructs. The six peptide toxins were recovered as predominant low-molecular-mass products, whereas PIGA was obtained as an electrophoretically heterogeneous preparation containing multiple His-immunoreactive species. These recombinant preparations were subsequently used for bioactivity screening.

### 2.2. Injection Toxicity of the Insecticidal Candidates Against S. litura Larvae

**Figure 2:**
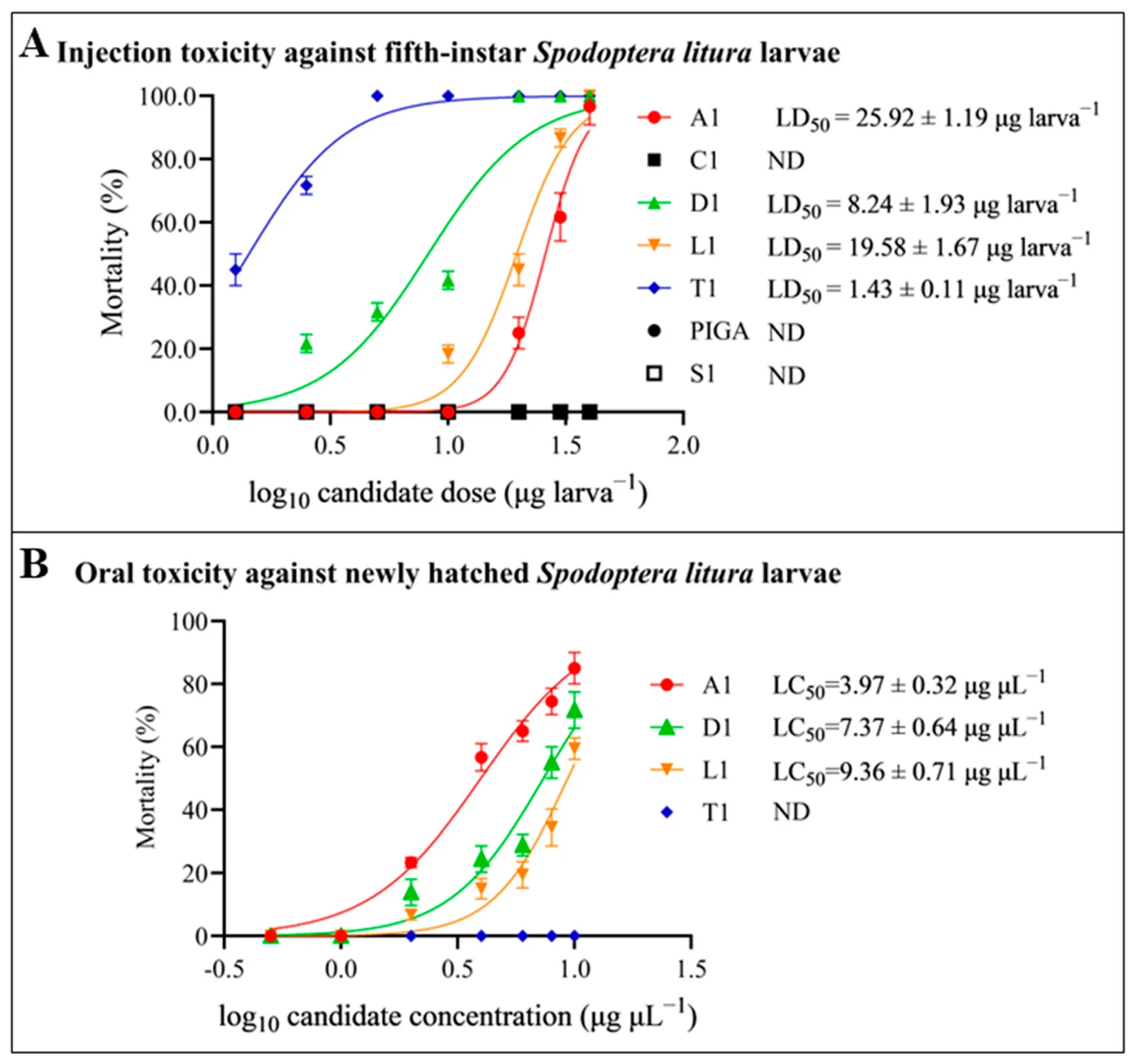
Injection and oral toxicity of recombinant insecticidal candidates against *Spodoptera litura* larvae. (**A**) Newly molted fifth-instar larvae were injected with graded doses of A1, C1, D1, L1, S1, T1, or PIGA, and mortality was assessed at 48 h post-injection. The LD_50_ values (mean ± SD) of T1, D1, L1, and A1 were 1.43 ± 0.11, 8.24 ± 1.93, 19.58 ± 1.67, and 25.92 ± 1.19 μg larva^−1^, respectively. C1, S1, and PIGA caused no mortality at doses up to 40 μg larva^−1^. (**B**) The four candidates with quantifiable injection lethality were subsequently evaluated using a leaf-disc feeding bioassay. Neonate larvae were fed leaf discs surface-treated with graded concentrations of A1, D1, L1, or T1, and mortality was assessed at 72 h after treatment. The LC_50_ values (mean ± SD) of A1, D1, and L1 were 3.97 ± 0.32, 7.37 ± 0.64, and 9.36 ± 0.71 μg μL^−1^, respectively. No mortality was observed following oral exposure to T1 at concentrations up to 10 μg μL^−1^. Solid lines represent the fitted dose–mortality curves in (**A**) and concentration–mortality curves in (**B**). Doses and concentrations are plotted on a log_10_ scale. Data points represent mean mortality, and error bars represent the SD. LD_50_, median lethal dose; LC_50_, median lethal concentration; ND, not determined.

The injection activity of the seven recombinant candidates was evaluated in newly molted fifth-instar *S. litura* larvae using a series of graded doses. Mortality at 48 h post-injection was used to generate dose–mortality curves and calculate median lethal doses (LD_50_; mean ± SD) (Figure 2A). At the maximum screening dose of 40 μg total protein per larva, the recombinant preparations generated from the C1, S1, and PIGA constructs produced no detectable mortality in fifth-instar *S. litura* larvae. In contrast, the A1, D1, L1, and T1 preparations exhibited measurable injection toxicity, with mortality increasing progressively with dose for each preparation, demonstrating dose-dependent lethality under the conditions tested.

T1 showed the greatest injection activity. At 5 μg larva^−1^, severe body twisting and segmental contraction appeared within 5 min (Video S1), and mortality reached 100% by 48 h. Dose–mortality analysis yielded an LD_50_ of 1.43 ± 0.11 μg larva^−1^ (Figure 2A). D1 produced similar acute intoxication signs, followed by death, and had an LD_50_ of 8.24 ± 1.93 μg larva^−1^ (Figure 2A). L1 also caused measurable injection lethality, with an LD_50_ of 19.58 ± 1.67 μg larva^−1^ (Figure 2A). Relative to T1, the mean LD_50_ values of D1, L1, and A1 were approximately 5.8-, 13.7-, and 18.1-fold higher, respectively.

A1 had the highest LD_50_ among the four injection-active peptides, at 25.92 ± 1.19 μg larva^−1^ (Figure 2A). Despite its comparatively weak lethal activity, a dose of 5 μg larva^−1^, corresponding to approximately one-fifth of the mean LD_50_, induced recurrent convulsions and intermittent tremors. This phenotype differed from the severe body twisting and segmental contraction elicited by T1, D1, and L1 (Video S2). Qualitative observations indicated that the A1-induced motor disturbances became more severe as the dose increased and persisted in surviving larvae for up to 48 h. The contrast was particularly evident at 5 μg larva^−1^, at which T1 caused complete mortality within 48 h, whereas A1 primarily produced sustained convulsions and tremors. These observations indicate that A1 and T1 differed not only in lethal activity but also in the nature and progression of the intoxication signs they induced.

Based on the 48-h LD_50_ values, injection activity followed the order T1 > D1 > L1 > A1, whereas no lethal activity was detected for C1, S1, or PIGA at doses up to 40 μg larva^−1^. The four candidates displaying measurable injection lethality were subsequently advanced to oral activity testing.

### 2.3. Oral Insecticidal Activity of Four Candidates Against S. litura in a Leaf-Disc Bioassay

The four peptides displaying quantifiable injection lethality were subsequently evaluated for oral activity against neonate *S. litura* larvae. A1, D1, L1, and T1 were tested using a leaf-disc coating bioassay adapted from Pinto et al. [21]. Pepper leaf discs were treated with graded peptide concentrations and infested with neonate larvae, after which mortality and visible feeding damage were assessed at 72 h (Figure S3A). Concentration–mortality curves were generated, and median lethal concentrations were estimated (Figure 2B).

Despite showing the greatest activity following injection, T1 produced no detectable oral lethality at concentrations up to 10 μg μL^−1^. Its LC_50_ could therefore not be estimated within the tested range and was reported as >10 μg μL^−1^ (Figure 2B). Leaf-disc damage was also comparable to that in the buffer-treated control, with no discernible protective effect . Thus, the strong injection activity of T1 did not translate into detectable oral efficacy under the conditions tested. D1 and L1 both caused concentration-dependent mortality following ingestion (Figure 2B). The LC_50_ values against neonate larvae were 7.37 ± 0.64 μg μL^−1^ for D1 and 9.36 ± 0.71 μg μL^−1^ for L1. At the highest concentration tested, 10 μg μL^−1^, D1 and L1 produced mean mortalities of 71.67% and 59.44%, respectively, although neither peptide caused complete mortality. These results established measurable oral activity for both candidates, with D1 showing greater activity than L1.

A1 showed the opposite activity profile across the two exposure routes. Although it ranked last among the four peptides in the injection assay, it produced the strongest oral response. Mortality increased progressively with A1 concentration, yielding an LC_50_ of 3.97 ± 0.32 μg μL^−1^ (Figure 2B). The mean LC_50_ values of D1 and L1 were approximately 1.9- and 2.4-fold higher, respectively, than that of A1, indicating that lower concentrations of A1 were required to elicit an equivalent lethal response. Visible feeding damage also declined as the A1 concentration increased (Figure S3B,C). Leaf discs in the buffer-treated control were extensively consumed, with large areas of tissue removed and numerous irregular perforations. At 2 μg μL^−1^, more of the leaf-disc area remained intact, although conspicuous feeding injury persisted. Damage was further reduced at 4 and 6 μg μL^−1^, where tissue loss was largely confined to localized perforations. At 8 μg μL^−1^, most leaf discs remained intact and exhibited only minor feeding scars. These representative phenotypes qualitatively paralleled the concentration–mortality response and indicated progressively greater preservation of leaf tissue with increasing A1 concentration.

The ranking of the candidates therefore differed markedly between exposure routes. Oral activity followed the order A1 > D1 > L1, whereas T1 showed no detectable effect within the tested concentration range. T1 ranked first following injection but lacked oral efficacy, while A1 combined the weakest injection lethality with the strongest oral activity. Within this candidate panel, injection activity therefore did not predict insecticidal efficacy following ingestion. Based on its comparatively low LC_50_ and concentration-associated preservation of leaf tissue, A1 was selected for high-cell-density fermentation and subsequent whole-plant foliar application assays.

### 2.4. High-Cell-Density Fed-Batch Fermentation and Purification of Recombinant A1

A1 exhibited the strongest oral activity among the four candidates evaluated in the leaf-disc bioassay, as indicated by its lowest LC_50_, and was therefore selected for scale-up production. The A1 transformant showing the highest apparent secretion level during the initial screen was cultivated using a high-cell-density fed-batch process adapted from established *P. pastoris* fermentation protocols [27,30,31].

**Figure 3:**
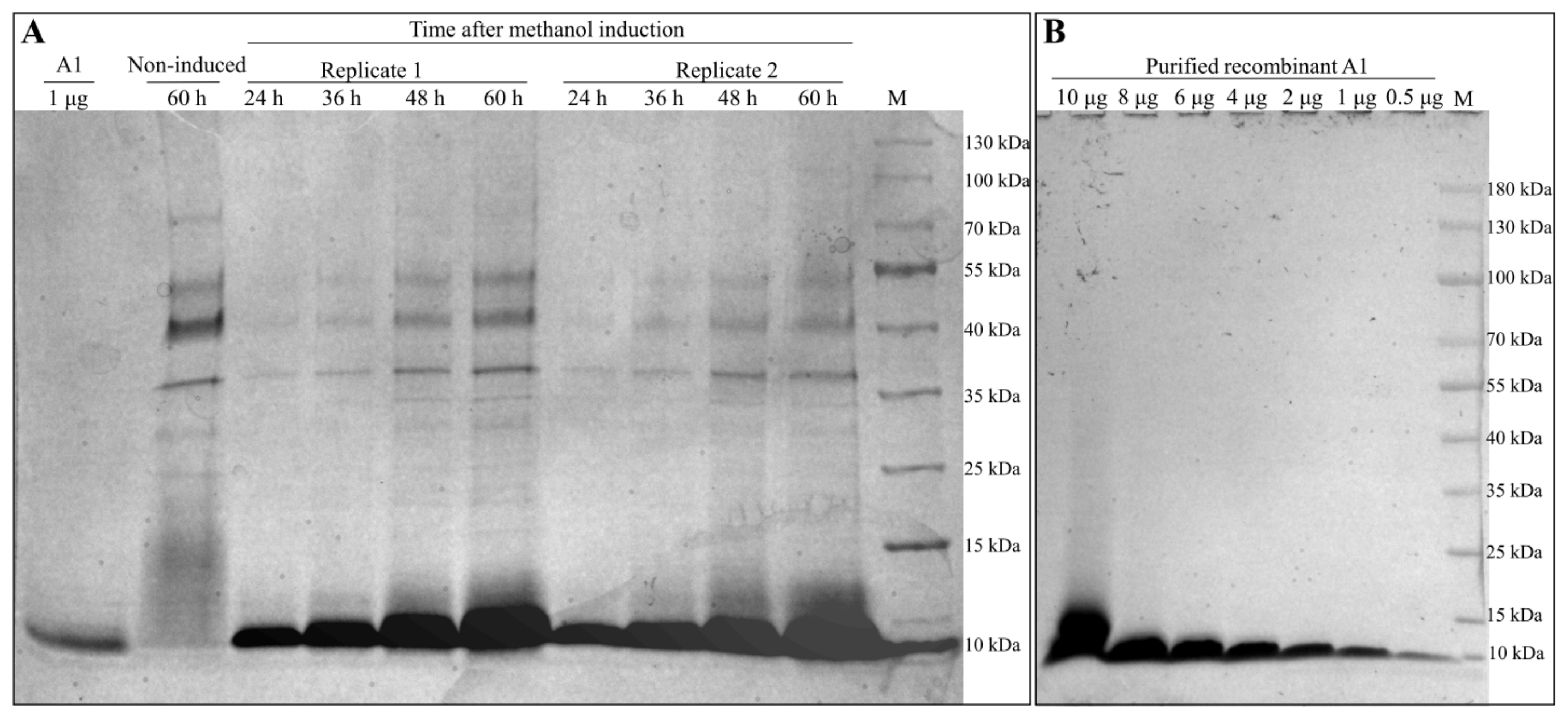
High-cell-density fermentation and two-step purification of recombinant A1. (**A**) SDS-PAGE analysis of recombinant A1 secretion during high-cell-density fermentation of *P. pastoris*. Culture supernatants were collected at 24, 36, 48, and 60 h after methanol induction. The two 24–60 h lane series represent duplicate technical loadings of the same time-course samples. Ten microliters of each culture supernatant was loaded per sample lane, and purified recombinant A1 (1 μg) was included as a reference. The non-induced control was culture supernatant collected after 60 h of cultivation in the absence of methanol. (**B**) SDS-PAGE analysis of recombinant A1 after sequential purification by nickel-affinity and ion-exchange chromatography. Purified A1 was loaded at 0.5, 1, 2, 4, 6, 8, and 10 μg per lane. M, molecular mass marker. Gels were stained with Coomassie Brilliant Blue.

Culture supernatants collected at 24, 36, 48, and 60 h after the onset of methanol induction were analyzed by SDS–PAGE. A distinct band co-migrating with the 1 μg purified A1 reference was already detectable at 24 h, and its staining intensity increased progressively through 60 h (Figure 3A). In contrast, the non-induced control collected at 60 h lacked this discrete band and showed only diffuse background staining in the corresponding molecular-mass region. These profiles indicated that accumulation of the A1-associated product was dependent on methanol induction and continued throughout the 60-h induction period. At 60 h, the total extracellular protein concentration was determined by BCA assay, and the relative abundance of the A1-associated band was assessed by ImageJ densitometry, with 1 μg of purified A1 serving as an external reference. Combining these measurements yielded a semiquantitative estimate of approximately 1.2 g L^−1^ for recombinant A1.

The fermentation supernatant was subsequently subjected to nickel-affinity chromatography followed by cation-exchange chromatography. When 0.5–10 μg of the two-step-purified preparation was analyzed by SDS–PAGE, a predominant band at approximately 10 kDa was observed across the entire loading range (Figure 3B). The intensity of this band increased with the amount loaded, whereas its migration position remained unchanged. No prominent additional protein bands were detectable under the Coomassie-staining conditions used. ImageJ densitometric analysis indicated that the predominant A1-associated band accounted for more than 99% of the summed intensity of all detectable bands, corresponding to an apparent electrophoretic purity exceeding 99%. The consistent migration of this band with the previously His-verified A1 product supported its assignment as the principal protein species in the final preparation. The two-step purification procedure therefore yielded recombinant A1 with high apparent electrophoretic purity.

Overall, under the fed-batch conditions tested, recombinant A1 reached an estimated titer of approximately 1.2 g L^−1^ after 60 h of methanol induction. Sequential nickel-affinity and cation-exchange chromatography provided sufficient material with an apparent electrophoretic purity exceeding 99% for the subsequent whole-plant foliar application assay.

### 2.5. Foliar Efficacy of A1 Against S. litura on Pepper Plants

To determine whether the activity observed in the leaf-disc assay translated to intact plants, the A1 preparation obtained by high-cell-density fermentation and two-step purification was evaluated under controlled whole-plant conditions. Graded concentrations of A1 were sprayed uniformly onto potted pepper plants, while control plants received the corresponding vehicle solution without A1. Once the foliage had air-dried, each plant was infested with three-day-old first-instar *S. litura* larvae. Visible feeding injury, larval mortality, and the body weight of surviving larvae were assessed 7 days after infestation (Figure S4).

**Figure 4:**
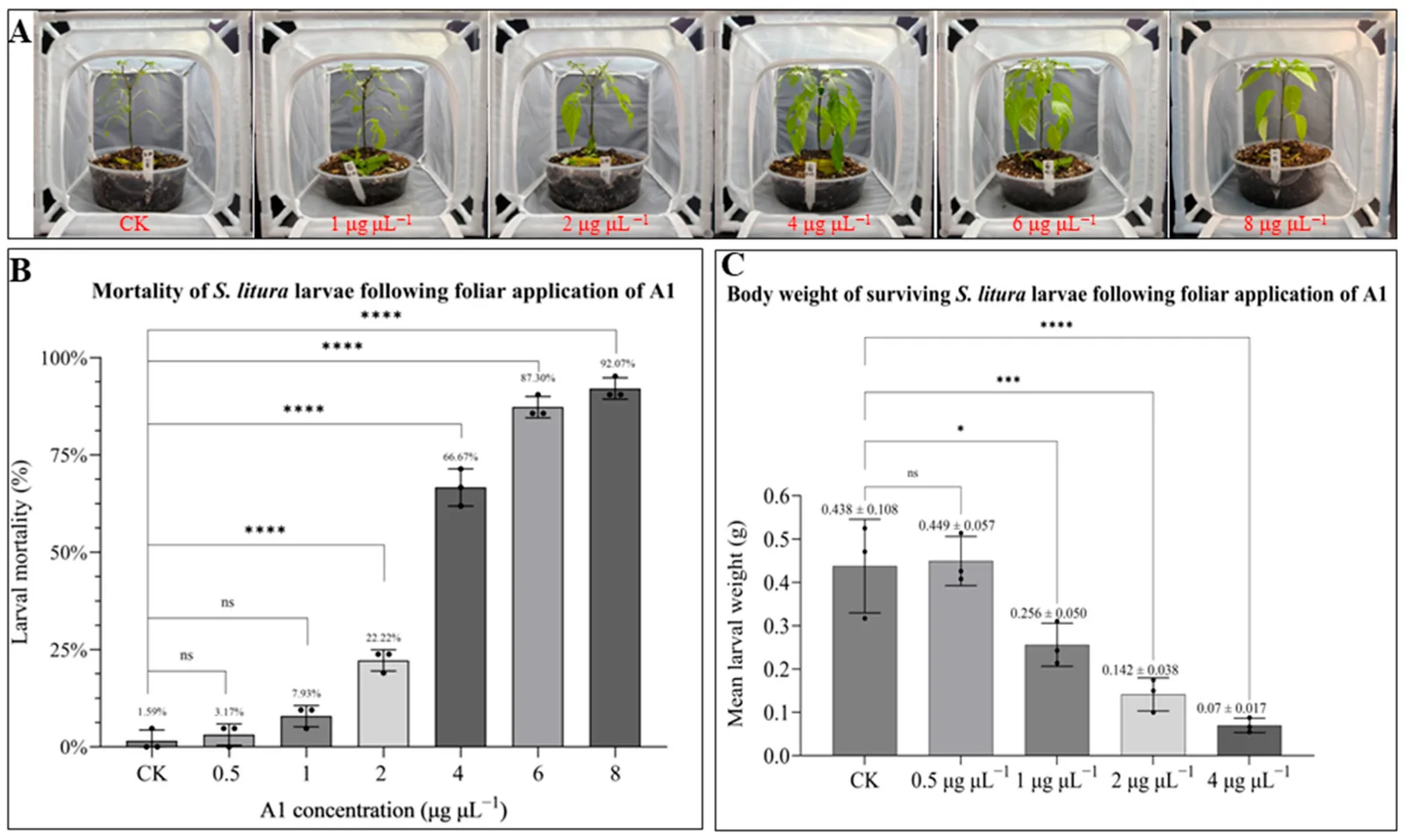
Concentration-dependent efficacy of foliar-applied A1 against *S. litura* larvae on pepper plants. (**A**) Representative whole-plant phenotypes recorded 7 days after infestation with 3-day-old first-instar *S. litura* larvae. Pepper plants were sprayed with the indicated concentrations of A1, and the spray deposits were allowed to air-dry before larval infestation. (**B**) Larval mortality at 7 days after infestation. (**C**) Mean body weight of surviving larvae at 7 days after infestation. Data in (**C**) are presented as the mean ± SD of three independent experimental replicates, with each dot representing one replicate. Statistical significance was evaluated by one-way ANOVA followed by Student’s *t*-tests comparing each A1 treatment with the vehicle control. ns, not significant; * *p* < 0.05; *** *p* < 0.001; **** *p* < 0.0001. CK, vehicle control without A1. All spray solutions, including CK, contained 0.1% (*v*/*v*) Silwet-L77.

Representative plant phenotypes showed progressively less feeding injury as the A1 concentration increased (Figure 4A). Larvae on vehicle-treated plants consumed most of the foliage, resulting in severe defoliation by 7 days after infestation. Considerable damage remained at the lower A1 concentrations, although the proportion of intact leaf tissue increased with spray concentration. At 4 μg μL^−1^, feeding injury was largely localized and the overall leaf structure was retained. Damage declined further at 6 and 8 μg μL^−1^, with leaves treated at 8 μg μL^−1^ remaining largely intact and displaying only minor feeding traces. These qualitative observations indicated a concentration-associated reduction in visible plant damage following foliar application of A1.

Larval mortality also increased with A1 concentration (Figure 4B). Mean mortality was 1.59% in the vehicle control, compared with 3.17% and 7.93% following treatment with 0.5 and 1 μg μL^−1^ A1, respectively. Neither treatment differed significantly from the control. Mortality rose to 22.22% at 2 μg μL^−1^ and 66.67% at 4 μg μL^−1^, reaching 87.30% and 92.07% at 6 and 8 μg μL^−1^, respectively. Every treatment at or above 2 μg μL^−1^ produced significantly greater mortality than the vehicle control, establishing a clear concentration-dependent lethal response to foliar-applied A1.

A1 exposure also reduced the body weight of surviving larvae (Figure 4C). Treatments at 6 and 8 μg μL^−1^ were excluded from this analysis because high mortality left too few survivors for reliable statistical comparison. The mean body weight was 0.438 ± 0.108 g in the control and 0.449 ± 0.057 g at 0.5 μg μL^−1^, with no significant difference between the two groups. At 1, 2, and 4 μg μL^−1^, mean survivor weights declined to 0.256 ± 0.050, 0.142 ± 0.038, and 0.070 ± 0.017 g, respectively, each significantly lower than the control value. These values represented reductions of 41.55%, 67.58%, and 84.02%, respectively. At 4 μg μL^−1^, surviving larvae attained only approximately 16% of the mean body weight recorded in the control.

Collectively, the three endpoints showed that A1 retained biological activity after deposition and drying on pepper foliage. Higher concentrations caused substantial mortality, whereas lower concentrations suppressed larval growth; both effects were accompanied by reduced visible feeding injury. These whole-plant findings support further evaluation of A1 as an active ingredient for foliar-applied peptide bioinsecticides.

## 3. Discussion

Herbivorous insect pests continue to impose substantial losses on global crop production, while the prolonged use of chemical insecticides has accelerated resistance evolution and raised concerns regarding residues, environmental contamination, and adverse effects on non-target organisms [32,33,34]. These limitations have stimulated interest in biologically derived insecticides with novel modes of action. Animal venoms and defensive secretions are particularly rich in insecticidal peptides, many of which possess disulfide-stabilized inhibitor cystine knot motifs that confer high structural stability and target selectivity [4,6,35]. The commercial development of ω/κ-HXTX-Hv1a-based insecticide illustrates the potential of venom peptides as crop-protection agents [17,36]. For foliar applications, however, intrinsic neurotoxicity alone is insufficient: an ingested peptide must resist degradation in the gut, traverse the midgut epithelium, and remain active after entering internal tissues [9,10]. Because these barriers vary among insect species and developmental stages, injection activity cannot be assumed to predict oral efficacy. This distinction is particularly relevant to *S. litura*, a highly destructive pest in which widespread insecticide resistance has increased the need for alternative control agents [3].

Accordingly, we evaluated seven previously reported insecticidal candidates using a sequential screening strategy. Injection assays were first used to determine whether the recombinant preparations retained activity after direct access to internal tissues, after which candidates with measurable injection lethality were advanced to oral testing. This route-specific comparison revealed a marked divergence between injection and oral activity. T1 was the most active candidate following injection but showed no detectable oral efficacy, whereas A1 exhibited the weakest injection lethality among the four injection-active peptides yet the strongest oral activity. Subsequent scale-up production and whole-plant assays further demonstrated that foliar-applied A1 caused larval mortality, suppressed the growth of survivors, and reduced visible feeding injury on pepper plants.

In the present study, the principal His-immunoreactive bands in all six peptide-toxin preparations migrated at apparent molecular masses higher than their calculated values on SDS–PAGE, whereas the PIGA preparation exhibited multiple His-immunoreactive species. Because these upward shifts and the heterogeneity of PIGA cannot be resolved by SDS–PAGE and anti-His immunoblotting alone, intact-mass spectrometry and LC–MS/MS peptide mapping would have been the preferred approaches for confirming sequence identity and characterizing molecular heterogeneity. In the absence of such analyses, however, the available indirect evidence supporting their assignment comprised several observations. First, all expression constructs were verified by DNA sequencing before transformation, and each candidate was expressed from a defined α-factor secretion cassette carrying an in-frame C-terminal 6×His tag. Second, the principal bands selected as putative products were enriched by nickel-affinity chromatography and coincided with the corresponding anti-His-immunoreactive signals. Third, several of the observed electrophoretic profiles resembled those previously reported for the same recombinant molecules or closely related disulfide-rich peptides expressed in *P. pastoris*. Recombinant nemertide α-1 (A1) was previously observed at approximately 10 kDa [24], recombinant LqhIT2 likewise migrated at approximately 10 kDa and was independently confirmed by nanoLC–MS/MS [19], and the heterogeneous electrophoretic profile of PI1a/GNA was previously investigated by N-terminal sequencing and PNGase F deglycosylation, supporting correct secretory processing and glycosylation-related heterogeneity [18]. Similar discrepancies between calculated and apparent molecular masses have also been reported for other *P. pastoris*-expressed ICK peptides [24,27]. Functional evidence provided a further, although indirect, line of support for the four injection-active preparations. A1, D1, L1, and T1 produced dose-dependent injection toxicity and characteristic intoxication phenotypes broadly consistent with their previously reported insecticidal or pharmacological properties. Taken together, construct verification, affinity enrichment, anti-His immunoreactivity, concordance with previously documented electrophoretic behavior, and, where present, biological activity support, but do not definitively establish, the assignment of the preparations as the intended recombinant products. Direct intact-mass spectrometry and LC–MS/MS analysis of the present preparations would be required for definitive molecular confirmation and for more precise determination of their exact masses, sequence coverage, and proteoform composition. The comparative bioactivity findings should therefore be interpreted in light of this limitation, particularly for C1, S1, and PIGA, which lacked detectable injection activity.

Among the seven candidates, CftICK-III (C1), U1-theraphotoxin-Spl1a (S1), and the PI1a/GNA fusion protein (PIGA) caused no detectable mortality in fifth-instar *S. litura* larvae following injection at doses up to 40 μg larva^−1^ and were therefore not advanced to oral testing. This lack of detectable mortality differs from previous reports in which C1 altered immune encapsulation and host survival in lepidopteran hosts of *Cotesia flavipes*, S1 exhibited injection and oral toxicity against *Helicoverpa armigera*, and PI1a acquired oral activity against *Mamestra brassicae* when fused to GNA [11,18,21]. For C1 and S1, these differences may reflect species- or stage-dependent sensitivity, variation in experimental conditions, or differences between the recombinant preparations examined here and the molecular forms evaluated previously. In the absence of direct molecular characterization, however, the relative contributions of these factors cannot be distinguished. The PIGA result warrants particular caution because, unlike the relatively simple electrophoretic profiles of the six peptide-toxin preparations, the affinity-purified PIGA preparation contained multiple His-immunoreactive species. Accordingly, the present findings should be interpreted as showing only that the specific C1, S1, and PIGA preparations tested here produced no detectable injection mortality at doses up to 40 μg larva^−1^ under the experimental conditions used; they do not establish that the corresponding molecules are intrinsically inactive against *S. litura*.

In contrast, A1, D1, L1, and T1 exhibited quantifiable injection lethality. T1 was the most active candidate, with an LD_50_ of 1.43 ± 0.11 μg larva^−1^, followed by D1 and L1, whereas A1 showed the weakest injection lethality. D1-, L1-, and T1-treated larvae developed similar acute intoxication signs, including pronounced body twisting and segmental contraction, followed by paralysis and death. D1 and L1 are insect-selective toxins that modify the activation of voltage-gated sodium channels (Na_V_) [8,37,38]. Their common effects on channel activation may account, at least in part, for the similar symptoms observed here, although their precise binding interactions are not necessarily identical. T1 activity has been associated with signaling mediated by insect nicotinic acetylcholine receptors, but its direct molecular target remains unknown [12].

Relative to the intoxication signs observed following treatment with the D1, L1, and T1 preparations, the A1 preparation produced a distinct intoxication profile in the present study. Although it had the highest LD_50_ among the four injection-active peptides, a dose of 5 μg larva^−1^ was sufficient to elicit pronounced convulsions and intermittent tremors. These symptoms became more severe with increasing dose and persisted in surviving larvae. A1 has previously been shown to delay the fast inactivation of insect Na_V_ channels, resulting in sustained sodium influx and neuronal hyperexcitation [23]. This effect provides a plausible explanation for the convulsive phenotype observed in *S. litura* and distinguishes A1 functionally from D1 and L1, which primarily alter channel activation. However, the binding site of A1 has not been conclusively established, and direct electrophysiological studies in *S. litura* would be required to link the observed symptoms to a specific molecular interaction.

Previous work demonstrated the oral activity of recombinant A1 against *M. brassicae*, *M. persicae*, and *A. pisum* [24]. The present study provides evidence that the oral activity of A1 also extends to *S. litura* and, by evaluating four candidates through both injection and oral exposure, reveals marked route-dependent differences in their relative performance. Injection assays bypass the digestive tract and midgut epithelial barrier and therefore more directly assess toxicity after a peptide gains access to the hemocoel, whereas oral bioassays additionally assess its ability to withstand digestion, traverse the midgut epithelium, and remain biologically active within internal tissues. Oral activity is therefore a more directly relevant criterion when evaluating peptides intended for conventional foliar application. Despite being the most potent candidate following injection, T1 caused no mortality at the highest oral concentration tested, 10 μg μL^−1^, and did not visibly reduce feeding damage to the leaf discs. D1 and L1 produced concentration-dependent mortality following ingestion, with LC_50_ values of 7.37 ± 0.64 and 9.36 ± 0.71 μg μL^−1^, respectively, although neither peptide caused complete mortality at the highest concentration tested. In contrast, A1, the least potent peptide following injection, exhibited the greatest oral activity, with an LC_50_ of 3.97 ± 0.32 μg μL^−1^. Increasing concentrations of A1 were also accompanied by progressively less visible feeding damage to the leaf discs. Thus, within the four candidates evaluated by both routes, the rank order of injection activity did not mirror their relative efficacy following ingestion. The absence of oral activity in T1 is particularly noteworthy. Davis et al. identified Arg9 of Ta1b as a major cleavage site for trypsin-like proteases in the lepidopteran gut and demonstrated that replacing this residue with glutamine increased proteolytic stability and conferred oral activity in selected test insects [12]. Nevertheless, the R9Q variant evaluated here remained orally inactive against *S. litura*. Stabilization of a single protease-sensitive site is therefore insufficient to ensure oral activity across different insect species. Residual proteolytic cleavage sites, inefficient transepithelial transport, instability or rapid clearance after entry into the hemocoel, and differences in target accessibility or sensitivity may all restrict oral efficacy. Furthermore, the composition and substrate specificity of midgut proteases differ among insect species. Consequently, a stabilizing mutation that is effective in one lepidopteran species may not provide equivalent protection in *S. litura*.

The biological origin of A1 offers one possible explanation for its comparatively strong oral activity. A1 is secreted into the epidermal mucus of the bootlace worm *Lineus longissimus* rather than being delivered directly into prey through fangs or a stinger. Molecules present in such external secretions are more likely to encounter prey through surface contact or ingestion, and this ecological context may favor physicochemical properties that promote persistence under external or digestive conditions. Long-term selection may therefore have enhanced the capacity of A1 to tolerate variable pH conditions, resist proteolytic degradation, or remain intact during passage across biological barriers. This interpretation remains hypothetical and will require direct measurements of digestive stability and midgut transport. Nevertheless, the present findings suggest that defensive mucus and other external secretions may represent particularly productive sources of peptide scaffolds for the development of foliar bioinsecticides.

In addition to biological activity, the practical development of peptide insecticides depends on their manufacturability. The present study, together with previous reports, further supports the utility of *P. pastoris* for producing disulfide-rich insecticidal peptides [19,24,27]. After 60 h of methanol induction during high-cell-density fermentation in a 10-L stirred-tank bioreactor, the A1-associated product reached a semi-quantitatively estimated titer of approximately 1.2 g L^−1^. This estimate was derived from BCA measurement of total extracellular protein and SDS–PAGE densitometry using purified A1 as a reference. Although this estimate is not equivalent to an absolute, product-specific analytical measurement, it is consistent with gram-per-liter-scale accumulation under the conditions tested. The fermentation also yielded sufficient material for downstream purification and whole-plant testing. These results provide preliminary evidence for the production potential of the current expression–fermentation platform and a basis for further process optimization and validated, product-specific quantification of titer and recovery.

The availability of sufficient purified material enabled the efficacy of foliar-applied A1 to be evaluated on intact pepper plants. Compared with detached leaf-disc assays, whole-plant assays provide a more application-relevant assessment because they preserve the physical architecture and physiological state of the host plant. Following application to pepper foliage, A1 remained biologically active and produced a clear concentration-dependent protective effect. Larval mortality reached 22.22%, 66.67%, 87.30%, and 92.07% at 2, 4, 6, and 8 μg μL^−1^, respectively, with all four treatments resulting in significantly greater mortality than the control. A1 also strongly inhibited the growth of surviving larvae. At 1 μg μL^−1^, mortality was not significantly elevated, yet the mean body weight of surviving larvae was reduced by 41.55%. Mean body weight was further reduced by 67.58% and 84.02% at 2 and 4 μg μL^−1^, respectively. Visible damage to pepper foliage decreased concurrently with increasing A1 concentration. This response pattern indicates that the plant-protective effect of A1 was not attributable solely to direct mortality. At concentrations that caused limited mortality, sublethal growth inhibition coincided with reduced visible foliage damage, suggesting that suppression of larval performance also contributed to plant protection.

The pharmacological action of A1 on Na_V_ inactivation provides a plausible explanation for both its lethal and growth-suppressive effects. At high exposure levels, sustained disruption of neuronal excitability may cause severe neuromuscular dysfunction and ultimately death. At lower exposure levels, persistent but nonlethal neuronal impairment may compromise larval locomotion and feeding, thereby restricting growth and reducing cumulative plant injury. The potential value of A1 for pest management may therefore arise from the combined effects of direct lethality and sublethal suppression of larval performance.

Taken together, our findings extend the previously reported oral activity of nemertide α-1 to the destructive polyphagous pest *S. litura*. Its concentration-dependent efficacy following application to intact pepper plants further supports its potential as an active ingredient for foliar-applied peptide bioinsecticides. Nevertheless, substantial challenges must be addressed before A1 can progress from laboratory validation to practical application, including production cost, formulation stability, field persistence, application efficiency, and consistency of performance under variable environmental conditions. Further development should therefore proceed along several complementary lines.

At the production level, multicopy expression cassettes could be assembled using approaches such as BglBrick cloning, followed by engineering of the *P. pastoris* secretory pathway and optimization of high-cell-density fed-batch fermentation. These measures could increase expression titers, improve batch-to-batch consistency and purification recovery, and reduce manufacturing costs. Molecular optimization could be guided by homology modeling, molecular dynamics simulations, and artificial intelligence-assisted sequence design to identify residues involved in the interaction between A1 and insect Na_V_ channels. Rational mutagenesis and directed evolution could subsequently be used to improve target affinity and insecticidal potency. Modifications intended to enhance stability in the insect gut and transport across the midgut epithelium, including fusion to cell-penetrating peptides or gut-targeting motifs, may further improve oral bioavailability.

Formulation and delivery will be equally important for practical application. Biocompatible carriers based on chitosan, liposomes, or mesoporous silica could protect A1 from ultraviolet radiation, phyllosphere microorganisms, and digestive proteases, thereby extending its persistence on plant surfaces and within the insect gut. Appropriately designed carriers may also promote epithelial uptake through receptor-mediated or other endocytic pathways. At the field level, the compatibility of A1 formulations with *Bacillus thuringiensis*, entomopathogenic fungi, and other biological control agents should be investigated as a strategy for broadening the insecticidal spectrum and delaying the evolution of resistance.

More broadly, marine invertebrates such as nemerteans remain an underexplored source of bioactive peptides with distinctive ecological functions and modes of action. Integrating multi-omics-based discovery, high-throughput activity screening, molecular engineering, and advanced delivery technologies should facilitate the identification of additional insecticidal molecules naturally suited to oral or external exposure. Such efforts could substantially expand the molecular resources available for sustainable agricultural pest management.

## 4. Materials and Methods

### 4.1. Microbial Strains, Vectors, and Reagents

Codon-optimized DNA sequences encoding the seven previously reported insecticidal candidates and the corresponding amplification primers were synthesized by General Biology Co., Ltd. (Chuzhou, China). *Escherichia coli* DH5α competent cells and horseradish peroxidase (HRP)-conjugated mouse monoclonal antibody against the His tag were purchased from Sangon Biotech (Shanghai, China). Restriction endonucleases, DNA polymerase, and the In-Fusion HD Cloning Kit were obtained from Takara (Otsu, Japan). The *Pichia pastoris* X-33 strain, the secretory expression vector pPICZαA, and Zeocin were obtained from Invitrogen (Carlsbad, CA, USA).

Prepacked HiPur Ni 6FF nickel-affinity columns (Cat. No. SA005C20) and HiPur SP 6FF cation-exchange columns (Cat. No. SI003C20) were purchased from Smart-Lifesciences Biotechnology Co., Ltd. (Changzhou, China). Centrifugal ultrafiltration devices with a molecular-weight cutoff of 3 kDa, filtration units, and 0.22 μm membrane filters were obtained from Millipore (Guangzhou, China). Silwet L-77 surfactant was purchased from General Electric Company (Fairfield, CT, USA). Unless otherwise specified, all other analytical-grade reagents were purchased from Biosharp (Hefei, China), Solarbio (Beijing, China), or Sangon Biotech (Shanghai, China). Low-salt LB, YPD, YPDS, BMGY, and BMMY media were prepared according to the instructions provided in the EasySelect™ *Pichia* Expression Kit manual (Cat. No. K1740-01; manual No. 25-0172; Invitrogen).

### 4.2. Construction of Recombinant Expression Plasmids and Transformation of Pichia pastoris

The pPICZαA expression cassettes were assembled in the present study, whereas their encoded insecticidal moieties were derived from previously published sequences and designs. A1, C1, D1, L1, and S1 encoded the previously reported mature toxin sequences [8,11,19,21,23,28,29]; PIGA encoded the previously described PI1a–AAA–GNA fusion protein [18]; and T1 encoded the previously described R9Q variant of U1-AGTX-Ta1b [12]. The coding sequences were codon-optimized for expression in *P. pastoris* without altering their encoded amino acid sequences. Apart from the addition of a C-terminal 6×His tag for purification, no further amino acid substitution or fusion element was introduced in the present study. Each coding sequence was inserted immediately downstream of the α-factor secretion leader in the methanol-inducible vector pPICZαA using the In-Fusion HD cloning system [39]. The vector–insert junctions were configured so that proteolytic processing of the α-factor leader would yield the intended mature N termini without additional vector-derived residues. All completed expression constructs were verified by DNA sequencing before transformation.

The pPICZαA vector was linearized by double digestion at the selected cloning sites. The candidate coding sequences were amplified from their respective synthetic templates using gene-specific primers bearing 15–20-bp 5′ extensions homologous to the corresponding vector ends. The purified amplicons and linearized vector were assembled using the In-Fusion HD Enzyme Premix at the manufacturer-recommended vector-to-insert molar ratio. Following incubation at 50 °C according to the manufacturer’s instructions, aliquots of the assembly mixtures were introduced into competent *E. coli* DH5α cells. Transformants were selected on low-salt LB agar containing 25 μg mL^−1^ Zeocin. Candidate colonies were screened by colony PCR, and plasmids recovered from PCR-positive colonies were verified by Sanger sequencing. Only constructs containing error-free inserts in the correct orientation and reading frame were retained for yeast transformation.

To generate the corresponding yeast expression strains, each sequence-verified plasmid was linearized with SacI, purified, and introduced into electrocompetent *P. pastoris* X-33 cells (Invitrogen, Carlsbad, CA, USA, Cat. No. C18000) by electroporation. Electrocompetent cells were prepared essentially as described by Cregg et al. [40], with minor modifications. Briefly, X-33 cells were cultured to an OD_600_ of 1.3–1.5, harvested, washed repeatedly with sterile water, and resuspended in 1 M sorbitol. Electroporation was performed at 1.5 kV, 25 μF, and 200 Ω. Immediately after the electrical pulse, 1 M sorbitol was added, and the cells were allowed to recover without agitation at 30 °C for 1–2 h. The recovered cell suspensions were spread onto YPDS agar plates containing 500 μg mL^−1^ Zeocin and incubated at 30 °C until individual colonies appeared.

### 4.3. Transformant Screening, Shake-Flask Induction, and Product Verification

For each recombinant construct, 14 independent Zeocin-resistant colonies with robust growth were chosen at random and inoculated individually into YPD broth containing 500 μg mL^−1^ Zeocin. Each preculture then served as a 1% (*v*/*v*) inoculum for 3 mL of BMGY medium in a sterile 50-mL culture tube. Cultivation proceeded at 30 °C and 250 rpm until the OD_600_ reached approximately 6.0. The cells were collected at 3000× *g* for 5 min and resuspended in BMMY medium to an OD_600_ of 10.0. Methanol induction was carried out at 28 °C and 250 rpm, with absolute methanol added every 24 h to give a final concentration of 1% (*v*/*v*) after each supplementation.

After 72 h of induction, the cultures were centrifuged to remove the cells, and the resulting supernatants were filtered through 0.22-μm membranes. Equal volumes of the filtrates were subjected to 15% SDS–PAGE, with 30 μL loaded in each lane. The gels were stained using a rapid Coomassie Brilliant Blue G-250 staining reagent, and the putative target bands were quantified with ImageJ software (v1.54p) (National Institutes of Health, Bethesda, MD, USA). All cultures had been initiated at the same OD_600_, and equivalent volumes of supernatant were compared; therefore, the relative intensity of each candidate band was used to rank the transformants. The clone displaying the highest apparent secretion level for each construct was selected for preparative shake-flask production.

Western blotting was used to examine the recombinant products obtained by nickel-affinity chromatography as described in Section 4.5. The purified samples were separated by SDS–PAGE and electrotransferred onto a blotting membrane. After blocking and washing under standard conditions, the membrane was probed with an HRP-conjugated mouse monoclonal antibody against the 6×His tag at a dilution of 1:5000. Immunoreactive signals coinciding with the corresponding SDS–PAGE bands provided complementary support for the presence of C-terminally His-tagged recombinant products in the purified preparations.

### 4.4. High-Cell-Density Fed-Batch Fermentation of Recombinant A1

High-cell-density fed-batch fermentation of recombinant A1 followed a procedure adapted from Wu et al. [27]. For seed preparation, the A1 transformant showing the highest secretion level in the small-scale screen was inoculated into YPG medium containing 1% (*w*/*v*) yeast extract, 2% (*w*/*v*) peptone, and 2% (*w*/*v*) glycerol. The culture occupied no more than 20% of the nominal flask capacity and was incubated at 30 °C and 220 rpm until the late exponential phase, corresponding to an OD_600_ of approximately 6.0.

A 5% (*v*/*v*) inoculum of the seed culture was transferred to a 10-L stirred-tank bioreactor (T&J Bioengineering, Shanghai, China) containing 5 L of defined fermentation medium prepared according to Matthews et al. [31]. The temperature was held at 28 °C, while automated addition of aqueous ammonia maintained the pH at 6.0. Cascade adjustment of agitation and aeration kept dissolved oxygen at or above 30% saturation. During fermentation, the agitation rate ranged from 300 to 1000 rpm, and the aeration rate ranged from 1 to 2 vvm.

Glycerol served as the primary carbon source during the initial batch phase. Its depletion was marked by a pronounced increase in dissolved oxygen, which triggered the start of glycerol feeding. The feed contained 70% (*w*/*v*) glycerol and 1.2% (*v*/*v*) trace metal solution (TMS). Upon completion of the glycerol-feeding phase, methanol supplemented with 1.2% (*v*/*v*) TMS was supplied to induce recombinant expression. The methanol feed rate was maintained within a range of 5–10 mL h^−1^ and adjusted in response to online changes in dissolved oxygen and pH. Induction continued for 60 h.

Samples were withdrawn at 24, 36, 48, and 60 h after the initiation of methanol feeding. Cell-free culture supernatants were obtained by centrifugation and analyzed by Coomassie-stained 15% SDS–PAGE followed by Coomassie staining. Each time-point sample was analyzed two technical replicate lanes, with 10 μL of supernatant loaded per lane. Purified recombinant A1 (1 μg) was included on the same gel as an external calibration standard, and supernatant from a parallel uninduced culture was included collected at 60 h was included as a negative control. Gel images were analyzed using ImageJ software (National Institutes of Health, Bethesda, MD, USA). The integrated density of each A1-associated band was measured within a fixed-size region of interest after subtraction of the corresponding local background.

The apparent amount of the A1-associated product in each sample lane was estimated by single-point densitometric calibration against the purified A1 standard. Assuming a proportional staining response, the integrated density of the sample A1 band was divided by that of the 1 μg A1 standard and multiplied by 1 μg. The resulting estimate of A1 mass was divided by the 10-μL loading volume to calculate the A1 concentration, which was subsequently expressed in g L^−1^. In parallel, the total extracellular protein concentration of each supernatant was measured in triplicate using a bicinchoninic acid assay. The relative abundance of A1 in the extracellular protein was calculated by dividing the estimated A1 concentration by the corresponding total extracellular protein concentration and multiplying by 100. The A1 titer at each time point was reported as the mean of the duplicate technical determinations.

### 4.5. Chromatographic Purification of Recombinant Products

Before chromatography, culture broths from shake-flask induction or high-cell-density fed-batch fermentation were adjusted to pH 7.0 with sodium hydroxide. Centrifugation at 12,000 rpm for 20 min removed the yeast cells and insoluble material, after which the clarified supernatants were passed through 0.22-μm membrane filters.

Purification of the C-terminally His-tagged candidates began with a HiPur Ni 6FF nickel-affinity column equilibrated in binding buffer containing 20 mM sodium phosphate and 500 mM NaCl (pH 7.0). Once the clarified culture supernatant had been loaded, five column volumes of wash buffer containing 20 mM sodium phosphate, 500 mM NaCl, and 30 mM imidazole (pH 7.0) were applied to remove unbound and nonspecifically retained proteins. The bound products were then eluted with 20 mM sodium phosphate, 500 mM NaCl, and 500 mM imidazole (pH 7.0). Fractions containing the respective target products were pooled. Material intended for bioassays was desalted and concentrated using 3-kDa molecular-weight-cutoff ultrafiltration devices. Before lyophilization, the total protein concentration of each preparation was determined using a bicinchoninic acid (BCA) assay, and the corresponding sample volume was recorded. The preparations were then lyophilized and stored until use. Before preparation of the bioassay solutions, each lyophilized sample was reconstituted with sterile double-distilled water to its recorded pre-lyophilization volume. Working solutions were prepared by diluting the reconstituted stocks to the required concentrations.

The apparent electrophoretic purities of the preparations used in the injection and leaf-disc feeding assays were estimated from the Coomassie-stained 15% SDS–PAGE gel using ImageJ software (National Institutes of Health, Bethesda, MD, USA). Following background subtraction, the integrated densities of the target band and all detectable discrete contaminant bands in each lane were measured. Apparent purity was calculated by dividing the integrated density of the target band by the summed integrated densities of the target and contaminant bands and multiplying the resulting value by 100.

For recombinant A1 produced by high-cell-density fermentation, nickel-affinity purification was followed by cation-exchange polishing because the larger-scale fermentation generated a greater extracellular protein background than shake-flask cultivation. The pooled A1-containing eluate was dialyzed against 20 mM sodium phosphate buffer (pH 7.0), with three buffer changes, to remove imidazole and lower the ionic strength. It was then loaded onto a HiPur SP 6FF cation-exchange column pre-equilibrated with the same buffer. A wash containing 20 mM NaCl removed unbound and weakly retained components, whereas bound A1 was resolved with a linear gradient of 20–500 mM NaCl in 20 mM sodium phosphate buffer (pH 7.0). The A1-containing fractions were combined, concentrated, and desalted using 3-kDa molecular-weight-cutoff ultrafiltration devices. After desalting, the protein concentration of the final A1 preparation was determined using BCA assay. Electrophoretic purity was assessed by Coomassie-stained 15% SDS–PAGE followed by densitometric analysis using ImageJ. Apparent electrophoretic purity was determined using the same procedure described above, with the integrated density of the principal A1-associated band divided by the summed integrated densities of all detectable discrete bands.

### 4.6. Injection Toxicity Bioassay Against Fifth-Instar S. litura Larvae

A stable laboratory colony of *S. litura* maintained in-house provided the insects used throughout the study. Newly molted fifth-instar larvae of comparable size were selected for the injection bioassay and chilled on ice for 30 min before treatment to reduce movement during handling. Each recombinant candidate was diluted in 0.1× phosphate-buffered saline (PBS) to concentrations of 0.25, 0.5, 1, 2, 4, 6, and 8 μg μL^−1^; 0.1× PBS alone served as the vehicle control.

Injections were administered with a 10-μL microsyringe (Gaoge, Shanghai, China) fitted with a tapered needle measuring 9 mm in length and 0.26 mm in outer diameter. A 5-μL aliquot of the appropriate test solution was delivered into the hemocoel through the intersegmental membrane between the third and fourth abdominal segments. The concentration series therefore corresponded to doses of 1.25, 2.5, 5, 10, 20, 30, and 40 μg larva^−1^. Control larvae received 5 μL of 0.1× PBS.

For each dose, 20 larvae constituted one biological replicate, and three independent replicates were performed, giving a total of 60 larvae per dose. Immediately after injection, the larvae were returned to their routine rearing conditions and supplied with fresh food. Signs of intoxication were monitored throughout the 48-h observation period, and mortality was recorded at 48 h. A larva was considered dead if it failed to respond to gentle tactile stimulation and could not resume coordinated movement; completely rigid individuals were also included in the mortality count. The resulting data were used to construct dose–mortality curves and estimate the median lethal dose (LD_50_, μg larva^−1^). LD_50_ values are expressed as the mean ± standard deviation of three independent bioassays. Candidates with quantifiable injection lethality were subsequently evaluated in the leaf-disc feeding bioassay.

### 4.7. Leaf-Disc Feeding Bioassay of Oral Insecticidal Activity

The four candidates that produced quantifiable lethality in the injection screen—A1, D1, L1, and T1—were taken forward for oral evaluation using a leaf-disc surface-treatment assay adapted from Pinto et al. [21]. Each peptide was prepared in 0.1× PBS at nominal concentrations of 0.5, 1, 2, 4, 6, 8, and 10 μg μL^−1^. The vehicle control (CK) consisted of 0.1× PBS without peptide.

Fresh, fully expanded pepper leaves of comparable developmental status were selected from plants showing no visible disease symptoms or herbivore damage. Discs 15 mm in diameter were excised with a cork borer. The assay was assembled in 12-well cell-culture plates, with each well containing 1 mL of 1% (*w*/*v*) agar. Once the agar had solidified, one leaf disc was placed on its surface to maintain moisture and delay wilting. The exposed surface of each disc received 40 μL of the appropriate peptide solution, distributed as evenly as possible. Control discs were treated with the same volume of 0.1× PBS. All discs were left to air-dry at room temperature until no visible droplets remained.

Five newly hatched *S. litura* larvae were introduced into each well after the applied solution had dried. One 12-well plate constituted a biological replicate, corresponding to 60 larvae, and three independent plates were established for each concentration. Thus, 180 larvae were evaluated at every treatment level. The plates were held in a controlled-environment chamber under the routine conditions used for colony maintenance. Larval survival and feeding activity were monitored throughout the assay, with mortality scored 72 h after larval introduction. Individuals that neither responded to gentle tactile stimulation nor resumed coordinated movement were classified as dead. Feeding injury was documented photographically and evaluated qualitatively by comparing tissue loss and perforation among treatments.

The 72-h mortality data were fitted to concentration–mortality curves to estimate the median lethal concentration (LC_50_). LC_50_ values refer to the nominal concentration of the solution applied to the leaf surface and are expressed in μg μL^−1^. Estimates are reported as the mean ± standard deviation of three independent bioassays.

### 4.8. Whole-Plant Foliar Spray Bioassay of A1 Against Spodoptera litura

Whole-plant activity was evaluated using A1 obtained by high-cell-density fermentation and two-step chromatographic purification, with the assay design informed by previously described foliar-exposure bioassays [17]. Fresh A1 solutions containing 0.5, 1, 2, 4, 6, or 8 μg μL^−1^ were prepared on the day of application. To promote wetting and spreading over the foliage, Silwet L-77 was added to every solution at 0.1% (*v*/*v*). The carrier control (CK) contained an equivalent concentration of Silwet L-77 but no A1.

Uniform potted pepper plants approximately 20 days old, each bearing about five leaves and one apical bud, were selected for the experiment. Approximately 10 mL of the appropriate solution was applied to each plant with a handheld sprayer, ensuring thorough coverage of both the adaxial and abaxial leaf surfaces. The foliage was then allowed to air-dry at room temperature. Once no visible droplets remained, seven three-day-old first-instar *S. litura* larvae were placed on each plant.

Three plants and 21 larvae together constituted one biological replicate. Three independent replicates were established for each treatment, corresponding to nine plants and 63 larvae per A1 concentration. Infested plants were maintained for 7 days at 25–28 °C, 60–70% relative humidity, and a 16 h light/8 h dark photoperiod. Direct watering of the leaves was avoided throughout the exposure period to minimize removal of the foliar A1 deposits. When irrigation was needed, water was applied only to the potting substrate.

Seven days after infestation, the numbers of surviving and dead larvae were recorded for each replicate, and mortality was calculated relative to the number initially introduced. Larvae were classified as dead when they failed to respond to gentle tactile stimulation and could not resume coordinated movement. All survivors were recovered and weighed, and their mean body weight was calculated separately for each biological replicate. Whole-plant phenotypes were also photographed to document visible feeding injury, including defoliation, perforation, and localized tissue loss, and to compare the degree of protection provided by the different A1 concentrations.

Mortality and survivor body-weight data are presented as the mean ± standard deviation of three independent biological replicates. The overall effect of A1 concentration was assessed by one-way analysis of variance. When the analysis indicated a significant treatment effect, each A1 treatment was compared with the carrier control using a two-tailed Student’s *t*-test. Differences were considered significant at *p* < 0.05.

### 4.9. Statistical Analysis

Statistical analyses and figure preparation were carried out in GraphPad Prism 8.0 (GraphPad Software, San Diego, CA, USA). For the injection and leaf-disc feeding bioassays, doses or nominal treatment concentrations were log_10_-transformed, and mortality responses were fitted by nonlinear regression using a four-parameter logistic model with a variable slope. LD_50_ and LC_50_ values were derived from the corresponding fitted curves.

No extrapolation beyond the experimentally tested range was performed when mortality remained below 50% at the highest dose or concentration, or when no mortality occurred across the entire range. In such cases, the endpoint was reported as greater than the highest tested level, and the numerical LD_50_ or LC_50_ estimate was designated as not determined (ND).

For the whole-plant foliar spray assay, one-way analysis of variance was used to test the overall effects of A1 concentration on larval mortality and the mean body weight of surviving larvae. A significant overall result was followed by two-tailed Student’s *t*-tests comparing each A1 treatment with the carrier control. Figure annotations indicate non-significant differences as “ns”; one, two, three, and four asterisks denote *p* < 0.05, *p* < 0.01, *p* < 0.001, and *p* < 0.0001, respectively.

## 5. Conclusions

This study extends the documented oral insecticidal spectrum of recombinant nemertide α-1 (A1) to *S. litura* and demonstrates its concentration-dependent efficacy following foliar application to intact pepper plants. The two-tier screening strategy further showed that injection potency did not reliably predict oral efficacy within the tested candidate panel. High-cell-density fermentation yielded an estimated recombinant A1 titer of 1.20 g L^−1^, providing a basis for further production optimization. Foliar-applied A1 increased larval mortality, suppressed the growth of surviving larvae, and reduced plant damage. Together, these findings support further evaluation of A1 as a candidate active ingredient for foliar peptide-based bioinsecticides and suggest that marine invertebrate toxins warrant further exploration as potential sources of crop-protection molecules.

## Data Availability

The data presented in this study are available in article and Supplementary Materials. And the raw data supporting the conclusions of this article will be made available by the authors on request.

## Supplementary Materials

The following supporting information can be downloaded at: https://www.mdpi.com/article/10.3390/md24090310/s1, Table S1: Origin and sequence classification of the seven recombinant insecticidal candidates. Figure S1: Schematic organization and amino acid sequences of the seven previously reported insecticidal candidates expressed in this study. Figure S2: SDS-PAGE screening of recombinant *Pichia pastoris* X-33 transformants expressing seven insecticidal candidates. Figure S3: Leaf-disc coating bioassay workflow and representative feeding damage following oral exposure of *Spodoptera litura* larvae to A1. (A) Schematic workflow of the leaf-disc coating bioassay used to evaluate the oral insecticidal activity of the four injection-active candidates. Pepper leaf discs were surface-treated with graded concentrations of each candidate and subsequently infested with newly hatched *S. litura* larvae. Each concentration was tested using three independent 12-well plates, with five larvae placed in each well. Larval mortality and feeding damage were assessed at 72 h after infestation. (B) Representative 12-well plates containing leaf discs treated with 0.1× PBS (CK) or selected concentrations of A1 (1, 2, 4, 6, or 8 μg μL^−1^), photographed 72 h after infestation. (C) Representative individual leaf discs from the corresponding treatments, illustrating the concentration-associated reduction in feeding damage. CK, buffer-treated control (0.1× PBS). Figure S4: Experimental workflow for evaluating the foliar efficacy of A1 against *Spodoptera litura* larvae on pepper plants. Graded concentrations of A1 (0.5, 1, 2, 4, 6, and 8 μg μL^−1^) and an A1-free vehicle control were prepared, with all spray solutions containing 0.1% (*v*/*v*) Silwet-L77. Approximately 20-day-old pepper plants bearing five leaves and one apical bud were sprayed until the foliage was uniformly wetted. After the spray deposits had air-dried, each plant was infested with seven 3-day-old first-instar *S. litura* larvae and maintained for 7 days at 25–28 °C and 60–70% relative humidity under a 16:8 h (L) photoperiod. Visible plant damage, larval mortality, and the mean body weight of surviving larvae were evaluated at 7 days after infestation. The experiment was conducted with three independent replicates. CK, vehicle control without A1. Video S1: Rapid onset of body twisting, segmental contraction in fifth-instar *Spodoptera litura* larvae following injection of T1 at 5 μg larva^−1^; Video S2: Persistent convulsions and intermittent tremors in fifth-instar *Spodoptera litura* larvae following injection of A1 at 5 μg larva^−1^.
