# Supplementary material for: A Nemertean-Derived Peptide Toxin Exhibits Oral Insecticidal Activity Against Spodoptera litura
Source: Mar Drugs. 2026 Sep 4;24(9):310. doi: 10.3390/md24090310 (PMC13608833; doi:10.3390/md24090310)
Supplement: Supplementary file 1 [file marinedrugs-24-00310-s001.zip › Figure S1.pdf]

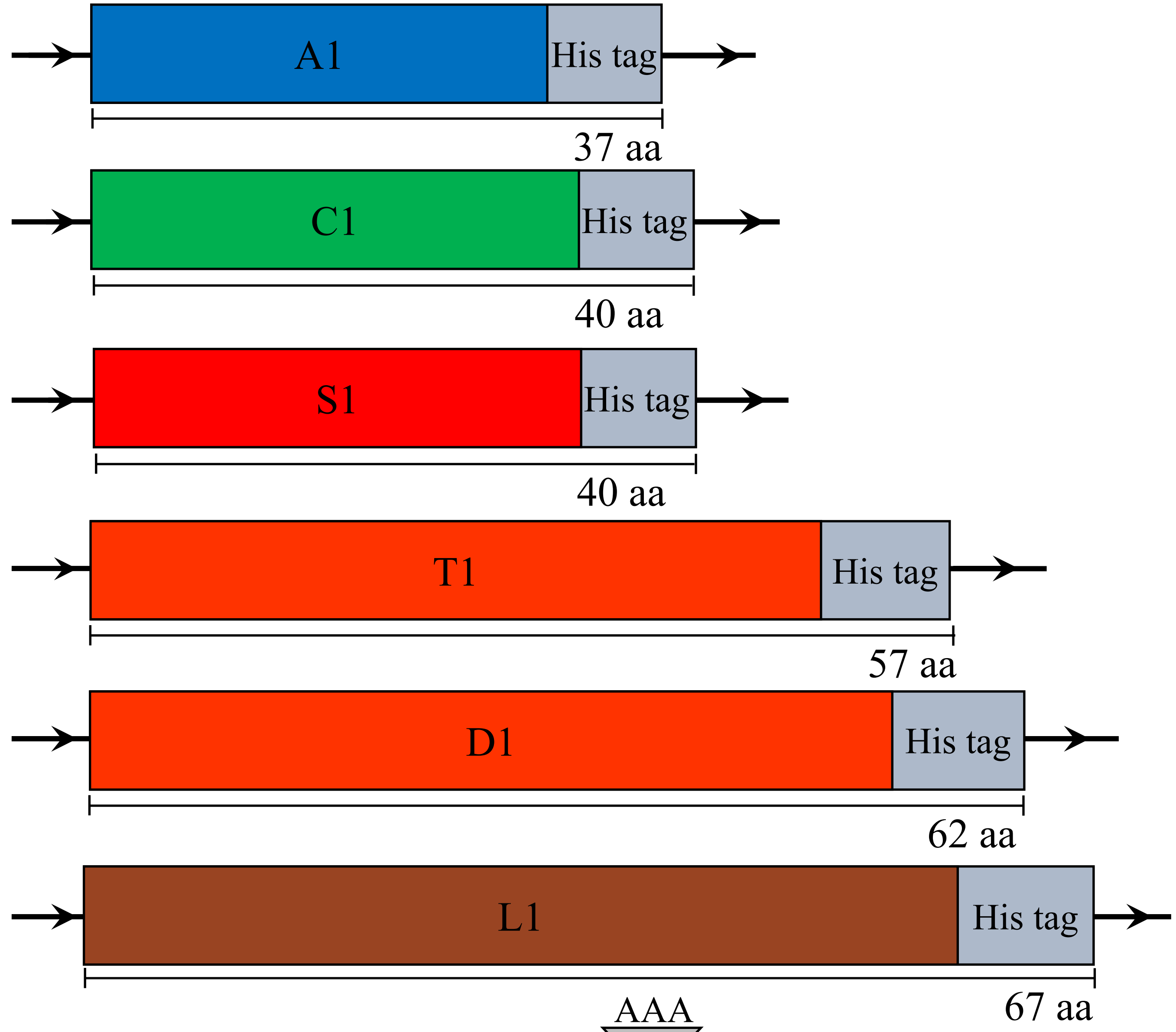

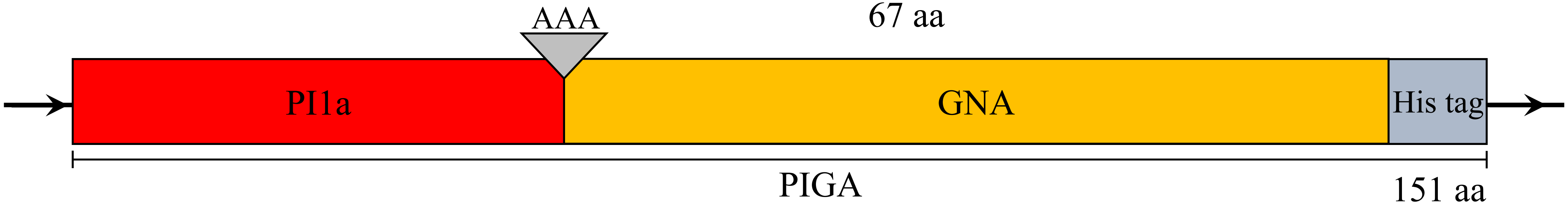

Construct	Sequence
A1	GCIATGSFCTLSKGCCTKNCGWNFKCNPPNQ
C1	DGCLTYDSLCSTMYDKCCTSMKCKGFFFGYCKSI
S1	DCGHLHDPCPNDRPGHRTCCIGLQCRYGKCLVRV
T1	EPDEICRAQMTNKEFTYKSNVCNNCGDQVAACEAECFRNDVYAACHEAQKG
D1	AKDGDVEGPAGCKKYDVECDSGECCQKQYLWYKWRPLDCRCLKSGFFSSKCVCRDV
L1	DGYIKRRDGCKVACLIGNEGCDKECKAYGGSYGYCWTWGLACWCEGLPDDKTWKSETNTCG
PIGA	GCLGEGEKCADWSGPSCCDGFYCSCRSMPYCRCRNNSAAADNILYSGETLSTGEFLNYGSFVFIMOEDCNLVLYDVDKPIWATNTGGLSRSCFLSMOTDGNLVVYNPSNKPIWASNTGGONGNYVCILOKDRNVVIYGTDRWATG
