# Supplementary material for: A Nemertean-Derived Peptide Toxin Exhibits Oral Insecticidal Activity Against Spodoptera litura
Source: Mar Drugs. 2026 Sep 4;24(9):310. doi: 10.3390/md24090310 (PMC13608833; doi:10.3390/md24090310)
Supplement: Supplementary file 1 [file marinedrugs-24-00310-s001.zip › Figure S2.pdf]

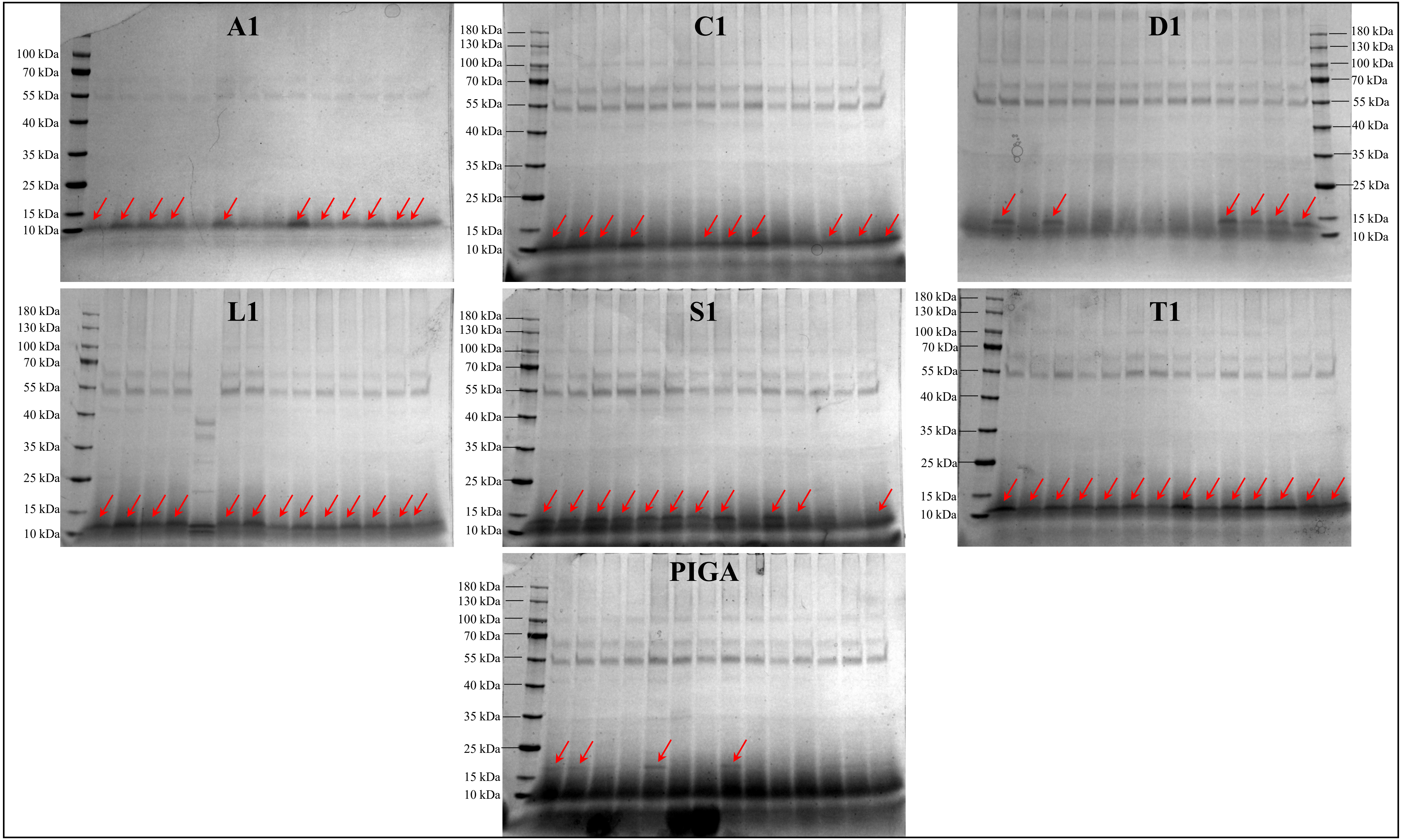
A1
100 kDa
70 kDa
55 kDa
40 kDa
35 kDa
25 kDa
15 kDa
10 kDa
C1
180 kDa
130 kDa
100 kDa
70 kDa
55 kDa
40 kDa
35 kDa
25 kDa
15 kDa
10 kDa
D1
180 kDa
130 kDa
100 kDa
70 kDa
55 kDa
40 kDa
35 kDa
25 kDa
15 kDa
10 kDa
L1
180 kDa
130 kDa
100 kDa
70 kDa
55 kDa
40 kDa
35 kDa
25 kDa
15 kDa
10 kDa
S1
180 kDa
130 kDa
100 kDa
70 kDa
55 kDa
40 kDa
35 kDa
25 kDa
15 kDa
10 kDa
T1
180 kDa
130 kDa
100 kDa
70 kDa
55 kDa
40 kDa
35 kDa
25 kDa
15 kDa
10 kDa
PIGA
180 kDa
130 kDa
100 kDa
70 kDa
55 kDa
40 kDa
35 kDa
25 kDa
15 kDa
10 kDa
