# Supplementary material for: A Nemertean-Derived Peptide Toxin Exhibits Oral Insecticidal Activity Against Spodoptera litura
Source: Mar Drugs. 2026 Sep 4;24(9):310. doi: 10.3390/md24090310 (PMC13608833; doi:10.3390/md24090310)
Supplement: Supplementary file 1 [file marinedrugs-24-00310-s001.zip › Figure S3.pdf]

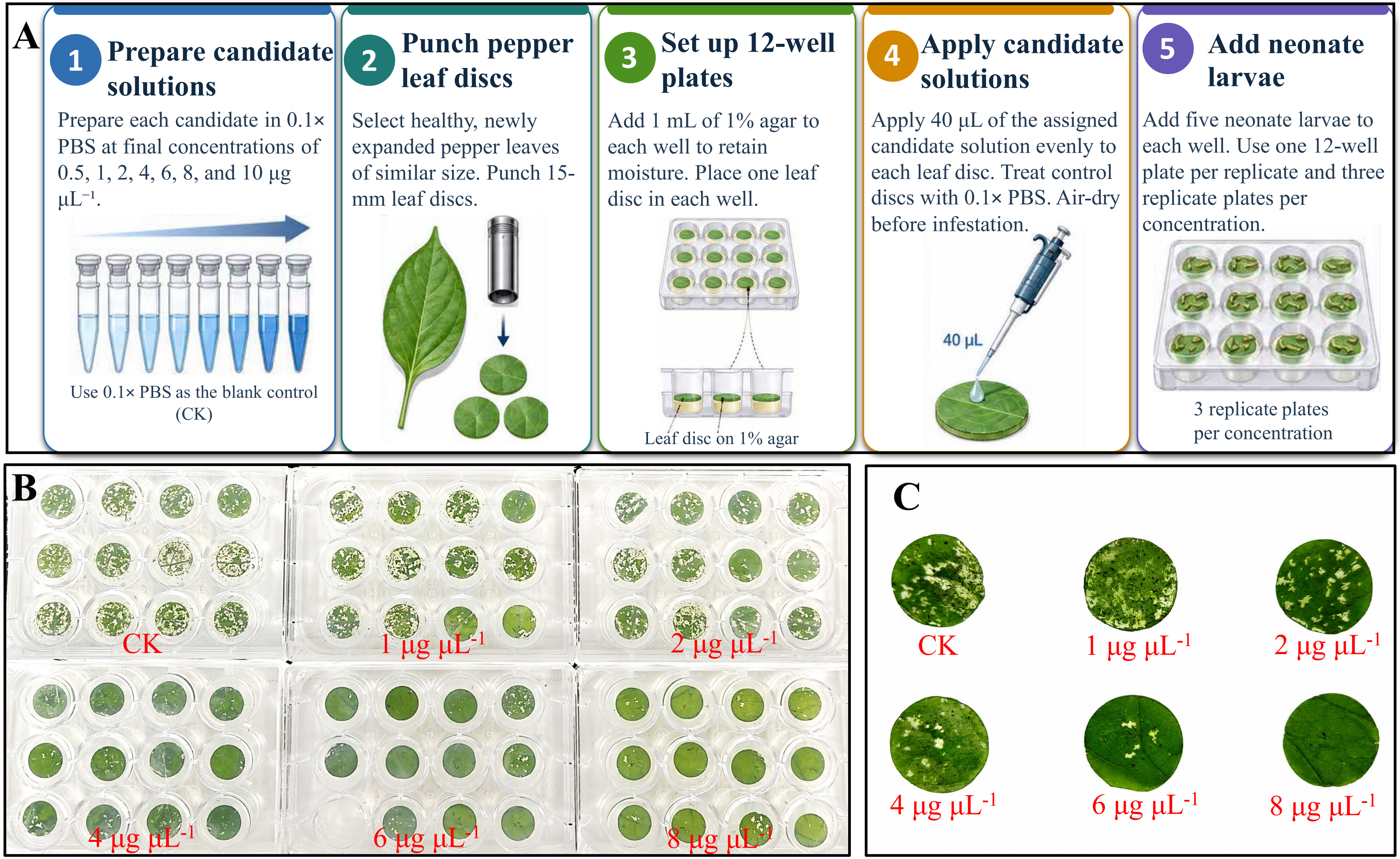
A
1 Prepare candidate solutions
Prepare each candidate in 0.1× PBS at final concentrations of 0.5, 1, 2, 4, 6, 8, and 10 µg µL⁻¹.
Use 0.1× PBS as the blank control (CK)
2 Punch pepper leaf discs
Select healthy, newly expanded pepper leaves of similar size. Punch 15-mm leaf discs.
3 Set up 12-well plates
Add 1 mL of 1% agar to each well to retain moisture. Place one leaf disc in each well.
Leaf disc on 1% agar
4 Apply candidate solutions
Apply 40 µL of the assigned candidate solution evenly to each leaf disc. Treat control discs with 0.1× PBS. Air-dry before infestation.
40 µL
5 Add neonate larvae
Add five neonate larvae to each well. Use one 12-well plate per replicate and three replicate plates per concentration.
3 replicate plates per concentration
B
CK
1 µg µL-1
2 µg µL-1
4 µg µL-1
6 µg µL-1
8 µg µL-1
C
CK
1 µg µL-1
2 µg µL-1
4 µg µL-1
6 µg µL-1
8 µg µL-1
