# Supplementary material for: A Nemertean-Derived Peptide Toxin Exhibits Oral Insecticidal Activity Against Spodoptera litura
Source: Mar Drugs. 2026 Sep 4;24(9):310. doi: 10.3390/md24090310 (PMC13608833; doi:10.3390/md24090310)
Supplement: Supplementary file 1 [file marinedrugs-24-00310-s001.zip › Figure S4.pdf]

1. Preparation of A1 Treatment Solutions

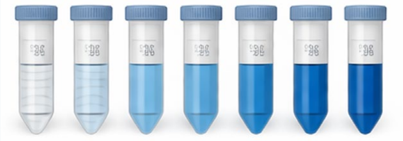

+0.1% Silwet-L77 (v/v) in all treatments.

2. Plant Preparation

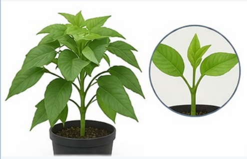

- Five fully expanded leaves and one apical bud;
- approximately 20 days old.

3. Foliar Application

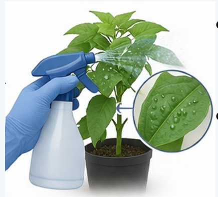

- Spray leaf surfaces until fully wet;
- Allow leaves to air-dry.

4. Infestation with *S. litura* Larvae

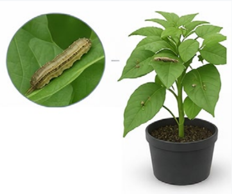

- First-instar larvae, 3 days post-hatching;
- 7 larvae per plant.

5. 7-day Incubation

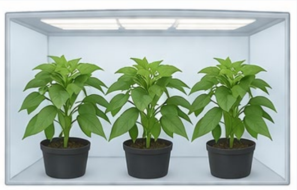

- 25–28 °C;
- 60–70% RH;
- 16 h light/8 h dark photoperiod.

6. Efficacy Assessment and Data Analysis

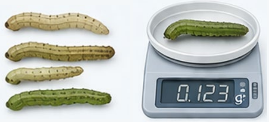

- Mortality (%) =(number of dead larvae / total number of larvae) ×100;
- Mean weight per surviving larva
